# Dental caries in 14- and 15-year-olds in New South Wales, Australia

**DOI:** 10.1186/1471-2458-13-1060

**Published:** 2013-11-09

**Authors:** John Skinner, George Johnson, Claire Phelan, Anthony Blinkhorn

**Affiliations:** 1 Centre for Oral Health Strategy NSW, 1 Mons Road, Westmead 2145, NSW, Australia; 2 Population Oral Health Unit, Faculty of Dentistry University of Sydney, 1 Mons Road, Westmead 2145, NSW, Australia; 3 South Eastern Sydney Local Health District, Locked Bag 21, Taren Point 2229, NSW, Australia

**Keywords:** Teen dental survey, School children, Adolescents, Caries experience

## Abstract

**Background:**

Dental caries remains one of the most common chronic diseases of adolescents. In Australia there have been few epidemiological studies of the caries experience of adolescents with most surveys focusing on children. The New South Wales (NSW) Teen Dental Survey 2010 is the second major survey undertaken by the Centre for Oral Health Strategy. The survey is part of a more systematic and efficient approach to support State and Local Health District dental service planning and will also be used for National reporting purposes.

**Methods:**

Data for the NSW Teen Dental Survey were collected in 2010 from a random sample of Year 9 secondary school students aged 14 to 15 years from metropolitan and non-metropolitan schools under the jurisdiction of the NSW Department of Education and Training, the Catholic Education Commission and Independent Schools in New South Wales. Nineteen calibrated examiners performed 1269 clinical examinations at a total of 84 secondary schools across NSW. The survey was accompanied by a questionnaire looking at oral health related behaviours, risk factors and the usage of the Medicare Teen Dental Plan.

**Results:**

175 schools were contacted, with 84 (48%) accepting the invitation to participate in the study. A total of 5,357 student consent forms and parent information packages were sent out and 1,256 students were examined; leading to a student participation rate of 23%. The survey reported a mean DMFT for 14 and 15 year olds of 1.2 and it was identified that 45.4% of students had an experience of dental caries. Major variations in caries experience reported occurred by remoteness, water fluoridation status, socio-economic status and household income levels.

**Conclusions:**

The NSW Teen Dental Survey provided state-wide data that will contribute to the national picture on adolescent oral health. The mean DMFT score of 1.2 is similar to the national caries experience data for this age group from the Australian Child Dental Health Survey in 2009.

## Background

Dental caries remains one of the most common chronic diseases of children and adolescents. For example in the United States dental caries is five times more common than asthma in children [1] with 59% of 12-19-year-olds having caries experience in their permanent teeth [2]. Similar levels of caries experience (40-57%) were recorded for 12-15-year-olds in the National Australian Child Dental Survey in 2003–04 [3]. The Australian Research Centre for Population Oral Health (ARCPOH) reported there is a lack of National dental caries data for teenage children aged 15–17 years in Australia [3]. This is particularly true in New South Wales, Australia’s most populous State, where there has been limited epidemiological surveys completed on adolescents. The main effort has been in measuring child dental health through the NSW Child Dental Health Survey in 2007, which examined the caries experience of five, and six year olds and 11 and 12 year olds [4].

Recent concerns have been raised about teenagers being at an increased risk of dental disease and dental caries experience, despite several decades of improvement in the dental health of children in Australia [5]. A study of teenage oral health in South Australia found that caries experience had increased over a 10-year period between 1996 and 2006 [6]. This finding was cited by the Australian Dental Association who expressed concerns about an increase in dental disease nationally in this age group [7]. Adolescence is a key life-stage when oral health behaviours such as toothbrushing and diet choices should become habits. However it is also a period during which parental influence wanes and adolescents become more independent in terms of both their general and health behaviour. A study reported that adolescence is an important period for the development of regular dental visiting behaviour [8] which was one of the reasons for the introduction of a National Medicare Teen Dental plan in 2008, by the Australian Commonwealth Government [9]. The plan offers preventive dental services to eligible teenagers aged 12-17-years-old via a voucher that can be used in both private and public dental services and would encourage regular dental visiting.

The NSW 2010 Teen Dental Survey was undertaken as part of a more systematic approach to oral health data collection. The aim was to provide contemporary data on the caries experience of 14- and 15-year-olds in New South Wales by recording the dental caries experience of a random representative sample of Year 9 students. A secondary aim was to record the observed enamel fluorosis/ defects in this sample. The survey was a collaborative effort between the Centre for Oral Health Strategy NSW (COHS), the University of Sydney, the University of Adelaide, and the Australian Research Centre for Population Oral Health (ARCPOH).

## Methods

Data for the NSW 2010 Teen Dental Survey were collected from a random sample of enrolled Year 9 secondary school students aged 14 to 15 years under the jurisdiction of the NSW Department of Education and Training, the Catholic Education Commission and Independent Schools. Schools from within each of the eight former Area Health Services in NSW were selected using a multistage, stratified random sampling procedure, based proportionately on the total child population residing within each Area Health Service. In this sampling frame the schools are defined as the primary sampling units. Schools were also stratified by their level of relative disadvantage using the Socio-Economic Index For Areas (SEIFA) [10] based on the school postcode. Schools were stratified into metropolitan and non-metropolitan areas to ensure a representative sample was selected.

The initial weight for each participant was calculated as the inverse of their probability of being selected in the survey. Due to differential response rates by Local Health District, age and sex, the initial weights were adjusted to ensure the distribution of the sample reflected the regional population distribution of 14-15-year-olds in NSW. Within each of the eight strata, sub-strata were defined by Local Health District (14 regions), sex (male/female) and age (14- to 15-year-olds). Each sub-stratum (56 sub-strata) was linked to the estimated resident population (ERP) for that sub-stratum from the Australian Bureau of Statistics.

Self-reported family income data were provided by parents or guardians as part of the questionnaire that accompanied the consent form and was used as a measure of socio-economic status (SES).

The place of residence for each participant was used to create a remoteness variable using the Australian Bureau of Statistics classification [11] and the SEIFA index of relative socioeconomic disadvantage was also linked by the participant’s postcode to create another measure of SES. Aboriginal status was coded from parental responses on the consent form and the accompanying questionnaire.

In 2010, nineteen teams of calibrated dental therapists and data recorders undertook clinical examinations based on the coding protocol of the Child Dental Health Survey NSW 2007, which was developed in partnership with the ARCPOH and the University of Sydney [4]. While data were collected on the primary and permanent dentitions at both the tooth (dmft/DMFT) and surface (dmfs/DMFS) levels, only DMFT is reported, as most of the 14- and 15-year-olds only have permanent teeth and to allow for comparison with international literature. Observed enamel fluorosis/defects were recorded for both the central incisors (11 and 21) using the Thylstrup and Fejerskov (TF) Index [12]. Only data for the 11 are reported in this paper.

Standard equipment, including portable air syringe compressors, lighting and dental instruments were used to maximise inter-examiner reliability. Parents were informed of the outcome of the examination via a letter handed to the teenager in a sealed envelope. A principal (Gold Standard) survey examiner who conducted the training and calibration of the examination teams also completed inter-examiner reliability testing on a sub-sample of 72 participants.

Clinical examination data were entered directly into the laptop computers and Cardiff Teleform software [13] was used to extract the data from the returned consent and questionnaire forms. Caries experience data were analysed in SAS 9.2 [14] and were compared with the socio-demographic data from the questionnaire and consent form using cross-tabulations along with ICC analysis and Kappa statistics for the reliability analysis. The key dependent variables used in the analyses were caries experience (DMFT > 0) and mean DMFT. The TABULATE and SURVEYFREQ procedures in SAS were used to produce cross-tabulations and determine 95% confidence intervals. The Chi-square test was used to assess the degree of association between water fluoridation status and enamel fluorosis/defects.

Ethics approval for the Survey was granted by the New South Wales Population and Health Services Research Committee and through the State Education Research Process (SERAP) of the New South Wales Department of Education and Training. The Catholic Education Commission and Association of Independent Schools also gave their permission to involve schools within their jurisdiction. SERAP number 2008279 and NSW Population & Health Services Research Ethics Committee, AU RED Reference: HREC/09/CIPHS/3.

## Results

175 schools were contacted, with 84 (48%) accepting the invitation to participate in the study. A total of 5,357 student consent forms and parent information packages were sent out via the schools. A total of 1,256 teenagers were examined including 591 males and 665 females (Table 1). The student participation rate was 23% which takes into account non-attendance and refusals on the day of the examinations.

**Table 1:** Dental caries status of 14 and 15 year olds, by sex, NSW 2010

**Sex**	**No. of children**	**Mean DMFT**	**Mean DMFT (DT > 0)**	**Mean DT**	**%DT > 0**
Males	591	1.2	3.1	0.5	24.4
Females	665	1.1	3.0	0.4	20.4
Total	1256	1.2	3.0	0.5	22.4

The examiner reliability was assessed by the gold standard examiner who re-examined 72 students (6% of all students examined). These students were also examined by one of the 14 survey examiners. Given the large distances involved it proved impossible to complete reliability examinations for five of the 19 examiners, who were responsible for assessing 12% of the total sample.

The 14 examiners whose clinical data were compared to the gold standard examiners reported high levels of agreement for tooth presence, missing, decayed or filled teeth, and for pre-cavitated lesions (ICC values ranged 0.8 to 0.9; Table 2). Excellent agreement was obtained for decayed, missing, filled or pre-cavitated status of individual teeth or surfaces and for TF scores (Kappa values ranged 0.7 to 0.9; Table 3).

**Table 2:** Kappa statistics for assessment of inter-rater reliability

**Index**	**No. of examiners**	**No. of teenagers**	**% agreement**	**Kappa**	**Weighted Kappa**
Enamel Fluorosis/defect category^*^	14	55	94.4	0.89	0.90
**Decayed, missing, filled or precavitated lesion category of individual teeth**	14	72	95.8	0.82	NA^#^
**Decayed, missing or filled category of individual teeth**	14	72	98.8	0.92	NA
**Decayed, filled or precavitated lesion category of individual surface**	14	72	98.8	0.77	NA
**Decayed or filled category of individual surface**	14	72	99.8	0.89	NA

^*^Teenagers were excluded if a non-fluorotic lesion was observed on the buccal surface or if a restoration or fixed orthodontic appliance was present on the labial coronal surface.

^#^NA – not applicable. Weighted Kappa values were only calculated for fluorosis index where the categories are ordinal.

**Table 3:** Intra-class correlations for assessment of inter-rater reliability

**Index**	**No. of examiners**	**No. of teenagers**	**ICC**
**Number of teeth present per teenager**	14	72	0.98
**Number of teeth missing due to pathology per teenager**	4	6	0.83
**Number of precavitated lesions per teenager**	13	34	0.84
**Number of decayed teeth per teenager**	12	25	0.89
**Number of filled teeth per teenager**	13	21	0.89
**Number of decayed, missing, filled or precavitated teeth per teenager**	14	53	0.88
**Number of decayed, missing or filled teeth per teenager**	13	39	0.93
**Number of decayed, missing, filled or precavitated surface per teenager**	14	53	0.90
**Number of decayed, missing or filled surface per teenager**	13	39	0.92

The mean DMFT for 14-and 15-year-olds was 1.2 with males having a slightly higher mean DMFT than females (1.2 versus 1.1) (Table 1). The overall mean decayed permanent teeth(DT) was 0.5 for males, and 0.4 for females (Table 1). Among 14- to 15-year-olds with untreated caries in their permanent teeth (DT > 0), the mean DMFT was 2.6 with an average of 1.0 decayed permanent teeth. Females had slightly higher DMFT than males (2.7 compared to 2.6).

Large differences were found among Local Health Districts, with Hunter New England having the lowest mean DMFT score of 0.5 teeth and Mid-North Coast having the highest at 3.0 teeth. There were also considerable differences in untreated caries rates between Local Health Districts with the lowest being 0.1 teeth in Sydney and the highest 1.6 teeth in the Mid-North Coast (Table 4). The overall percentage of 14- and 15-year-olds free from dental caries in their permanent teeth was 54.6%. However Table 2 shows that these percentages varied from a low of 30.8% in Mid-North Coast to a high of 72.8% in the Central Coast.

**Table 4:** Summary data on dental caries experience for 14-and 15-year-olds by NSW local health districts, 2010

**Local health district**	**No. children**	**Mean termanent teeth**	**Mean decayed teeth**	**% decayed teeth > 0**	**Mean missing teeth**	**% missing teeth > 1**	**Mean filled teeth due to caries**	**% filled teeth > 0**	**Mean DMFT**	**Percent caries free**	**% fissure sealant**
**Sydney**	78	27.5	0.1	8.0	0.03	3.4	0.5	29.4	0.7	62.8	36.8
**South Western Sydney**	144	27.6	0.6	31.9	0.05	2.7	0.7	26.3	1.3	48.6	12.6
**South Eastern Sydney**	92	27.5	0.2	12.3	0.00	0.0	1.2	46.2	1.4	48.8	51.7
**Illawarra Shoalhaven**	60	26.1	0.4	26.5	0.07	7.2	0.7	37.9	1.2	52.0	15.6
**Western Sydney**	72	27.7	0.8	29.5	0.04	1.1	0.8	35.6	1.7	44.3	23.2
**Nepean Blue Mountains**	87	27.4	0.3	18.0	0.06	2.2	0.8	34.5	1.2	56.1	36.4
**Northern Sydney**	94	27.6	0.3	17.8	0.01	1.3	0.5	21.5	0.8	63.6	33.7
**Central Coast**	25	27.1	0.3	20.7	0.00	0.0	0.1	12.6	0.5	72.8	35.2
**Hunter New England**	168	27.4	0.2	13.5	0.02	1.5	0.3	19.0	0.5	67.5	20.1
**Northern NSW**	112	27.4	0.7	39.3	0.05	2.7	1.1	43.4	1.9	37.3	38.8
**Mid-North Coast**	95	27.5	1.6	51.3	0.10	8.0	1.3	46.3	3.0	30.8	36.2
**Southern NSW**	42	27.7	0.2	9.6	0.04	4.1	0.4	20.9	0.6	71.2	38.8
**Murrumbidgee**	77	27.5	0.2	10.6	0.02	1.8	0.6	30.4	0.8	65.3	18.9
**Western NSW**	110	27.2	0.7	33.7	0.12	5.7	0.8	40.9	1.6	39.6	20.8
**NSW Total**	1256	27.4	0.5	22.4	0.04	2.4	0.7	30.6	1.2	54.6	28.1

The survey collected data on the presence of fissure sealants. The percentage of 14- to 15-year-olds with at least one fissure sealant present in their permanent teeth was 28.1% state-wide with the lowest percentage being 12.6% in South Western Sydney and the highest 51.7% in South-Eastern Sydney (Table 4).

There were 27 Aboriginal and/or Torres Strait Islander teenagers in the sample, comprising 2.1% of the survey population. There were insufficient data to provide reliable statistics on state-wide differences in dental caries experience among Aboriginal and non-Aboriginal children.

The teenagers whose parents were in the lowest income group experienced substantially higher rates of dental disease than those in the highest income group (1.8 versus 0.7 teeth; Table 5) and had on average twice as many teeth with untreated decay (0.9 versus 0.3 decayed teeth). Only 65 teenagers in NSW (5.2% of the survey population) required immediate treatment and recorded a mean DMFT score of 2.5 and a mean of 1.3 carious teeth (Table 5). These teenagers include those who were identified with existing tooth pain; abscessed, grossly decayed, avulsed or fractured teeth and severe periodontal problems. Those requiring immediate treatment had on average more than three times the number of untreated decayed permanent teeth (D = 1.3) than those individuals not requiring immediate treatment (D = 0.4).

**Table 5:** Weighted oral health indicators for 14 and 15 year old children by NSW population sub-groups, 2010

**Sub-groups**	**No. children**	**Mean permanent teeth**	**Mean decayed teeth (D)**	**% decayed teeth > 0**	**Mean missing teeth due to caries**	**% missing teeth > 1**	**Mean filled teeth (FT)**	**% filled teeth > 0**	**Mean DMFT**	**% caries free**
**Fluoridated areas**										
Un-fluoridated	249	27.2	0.8	33.7	0.06	3.9	0.8	34.8	1.7	45.0
Fluoridated	1007	27.5	0.4	20.7	0.03	2.1	0.6	30.0	1.1	56.0
NSW total	1256	27.4	0.5	22.4	0.04	2.4	0.7	30.6	1.2	54.6
**Immediate treatment needed**										
No immediate treatment needed	1185	27.4	0.4	19.5	0.03	1.9	0.6	29.7	1.1	57.1
Immediate treatment needed	65	27.1	1.3	72.9	0.15	11.0	1.1	46.5	2.5	9.6
NSW total	1250	27.4	0.5	22.4	0.04	2.4	0.7	30.6	1.2	54.5
**Socioeconomic Status**										
Most disadvantaged	279	27.2	0.7	30.3	0.07	4.8	0.7	30.8	1.5	49.2
Second most disadvantaged	307	27.5	0.4	21.6	0.03	2.6	0.6	34.1	1.0	52.0
Middle quintile	248	27.5	0.4	21.8	0.06	2.3	0.8	32.6	1.3	54.9
Second most advantaged	223	27.4	0.4	19.2	0.03	1.4	0.5	22.5	0.9	61.7
Most advantaged	199	27.6	0.3	19.4	0.01	0.7	0.7	33.0	1.1	55.1
NSW total	1256	27.4	0.5	22.4	0.04	2.4	0.7	30.6	1.2	54.6
**Income**										
Up to $20000	87	27.6	0.9	33.2	0.02	2.3	1.0	43.8	1.8	39.5
$20001-$40000	197	27.4	0.5	23.8	0.06	4.7	0.8	39.6	1.4	48.6
$40001-$60000	180	27.7	0.5	28.3	0.02	1.5	0.9	37.9	1.4	44.0
$60001-$80000	180	27.4	0.6	22.7	0.07	4.1	0.5	25.5	1.2	56.8
$80001-$100000	185	27.4	0.3	21.1	0.02	1.0	0.6	25.7	1.0	57.6
$100001-$120000	110	27.3	0.2	15.2	0.01	1.4	0.5	23.8	0.7	63.1
$120001 & above	181	27.4	0.3	13.2	0.01	0.2	0.4	21.4	0.7	69.0
NSW total	1120	27.5	0.5	22.1	0.03	2.2	0.7	30.7	1.2	54.7
**Remoteness**										
Major cities	628	27.4	0.4	21.9	0.03	1.7	0.7	30.8	1.2	54.8
Inner regional	507	27.5	0.5	23.3	0.05	3.4	0.6	29.8	1.2	55.4
Outer regional	107	27.2	0.5	21.6	0.03	2.9	0.7	30.5	1.2	51.0
Remote Australia	14	27.0	1.2	41.9	0.17	11.1	1.1	52.0	2.4	28.0
NSW total	1256	27.4	0.5	22.4	0.04	2.4	0.7	30.6	1.2	54.6

Teenagers living in fluoridated areas of NSW had lower mean DMFT rates (DMFT 1.1 versus 1.7, Table 5) and a higher percentage of children who had never experienced decay (56.0% versus 45.0%) than children in un-fluoridated areas.

Mean DMFT scores by socio-economic status (SES) were also compared, and these varied from 1.1 in the highest SES quintile to 1.5 in the lowest SES quintile (Table 5). Similarly, the mean number of decayed teeth varied from 0.3 in the highest SES quintile to 0.7 in the lowest SES quintile. The percentage of 14- and 15-year-olds with no caries experience was 55.1% in the highest SES quintile and 49.2% in the lowest SES quintile.

The mean DMFT showed an ascending gradient with increasing remoteness, with a mean score of 1.2 in the major cities and 2.4 in the remote areas (Table 5). The number of carious teeth (DT) showed a similar gradient, with an average of 0.4 in the major cities to 1.1 in remote areas. The percentage of teenagers with no caries experience varied from 54.8% in the major cities to 51.0% and 28.0% in the outer regional and remote areas, respectively.

The majority of 14- and 15-year-olds had no enamel fluorosis/defects, with 77% having TF scores of 2 and under (Table 6). There were no statistical differences in TF scores between fluoridated and un-fluoridated cohorts (χ^2^ = 4.89 (4df), P = 0.30).

**Table 6:** Enamel fluorosis/defect experience by water fluoridation status, 14–15 year olds

Enamel fluorosis/defect category*	**%**	**n**	***95% CI***
**Fluoridated**			
Normal (TF 0)	83.5	675	*80.0-87.0*
Barely detectable/ perceptible (TF 1, 2)	16.3	124	*12.8-19.7*
Mild (TF 3)	0.2	3	*0.0-0.3*
Moderate (TF 4, 5)	0.1	1	*0.0-0.3*
**Non-fluoridated**			
Normal (TF 0)	88.5	173	*83.4-93.6*
Barely detectable/ perceptible (TF 1, 2)	10.0	24	*6.4-13.7*
Mild (TF 3)	1.4	2	*0.0-3.6*
Moderate (TF 4, 5)	0	0	*-*
**NSW**			
Normal (TF 0)	84.1	848	*81.0-87.3*
Barely detectable/ perceptible (TF 1, 2)	15.4	148	*12.3-18.5*
Mild (TF 3)	0.3	5	*0.0-0.7*
Moderate (TF 4, 5)	0.1	1	*0.0-0.3*

*TF values refer to the Thylstrup and Fejerskov Index.

## Discussion

This is the first large epidemiological study of the dental caries experience of 14- and 15-year-olds in Australia using a population-based sampling methodology and calibrated examiners. The research design used the school as the sampling unit and in 2010/11 2631 children were reported to be taught at home [15], which is a very small portion (0.2%) of the total school-aged population. The design used in this study was developed in conjunction with ARCPOH and the University of Sydney. The sampling and examination protocol is almost identical to that being used in the 2012/2013 Australian National Child Dental Health Survey.

A potential weakness of this study is the low acceptance rate from schools and low participation rate of students. The acceptance rate from the schools was 48% and the reasons provided by the schools included a crowded curriculum, timetabling issues and prior commitments to other surveys. The participation rate from students was low at 23%, demonstrating the difficulty in acquiring consents from a teenage population sample. A previous New South Wales SOKS school dental program which ran from 1996 to 2000 [16] suffered similar problems. The survey involved Year 8 School grade (ages 12–13) students and achieved a participation rate of 40% for 12-13-year-olds compared to 72% of the kindergarten grade.

There were also only 27 (2.1%) Aboriginal participants who could be coded from their parents’ responses on the consent forms. The coding of Aboriginal status for both participants and their parents was also an issue with the data collected in the National Child Dental Health Surveys in 2003/04 [3] and in 2007 [4]. The lack of information about this priority group is disappointing given their normally high dental disease burden and the focus on closing the gap in Aboriginal oral health status being a major part of the National Oral Health Plan [17]. The lack of robust data on Aboriginality in recent surveys is likely to be addressed by the National Child Dental Health Survey 2012/2013.

The most recent nationally reported data for teenagers in Australia is from the 2009 Child Dental Health Survey Australia [18] which reported a mean DMFT of 1.7 for 14-year-olds, however no NSW data were available [18]. The BASCD survey of 14-year-olds in the United Kingdom found DMFT scores of 2.1 in 1990 and 1.7 in 1994 [19] while the 2009 New Zealand Oral Health Survey found a DMFT of 1.9 amongst 12-17-year-olds [20]. The most recent NSW data with adequate sample size and adjustment for under-estimating DMFT were collected in 2000 [21] and reported a mean score of 1.1 for 14-year-olds, which was similar to the mean DMFT of 1.2 for 14- and 15-year-olds in this 2010 survey, a decade later. The DMFT score has been stable in New South Wales over the last ten years and this may be due in part to the increased coverage of water fluoridation in the State over this time period [22].

The NSW Teen Dental Survey provides state-wide data on teenage children and is an important contribution to the national picture on teenage oral health. This survey also addresses the lack of data identified in the recent report on the dental health of Australian Teenagers [3]. The Child Dental Health Survey Australia 2007, noted difficulties in producing representative national estimate of caries experience for 13- to 15-year-olds. This was deemed due to differences in eligibility criteria for public dental services for this age-group among Australian States and Territories [4]. The Australia-wide National Child Dental Survey 2012/2013 currently underway includes 14- and 15-year-olds and will provide the first Australian National data based on a common sampling and examination protocol. However the NSW Teen Dental Survey has provided the first step in assembling a workable database of the oral health of teenagers and should enable health service planners and policy makers to make informed decisions.

Despite an overall mean DMFT score of 1.2, there are substantial differences within and among Local Health Districts in NSW and among sub-populations. This is particularly true for those teenagers living in un-fluoridated parts of NSW, those from lower SES backgrounds and those located in remote areas. These findings support the NSW Department of Health strategy to increase the proportion of the population receiving fluoridated public water supplies.

A critical issue is that 14-15-year-olds fall within a policy grey-area nationally in Australia. In some states 14-15-year olds are universally eligible for free public dental care whereas in other states they need to have parents who are health care card holders and/or pay a co-payment. This has meant that data collection has been historically limited to inspections undertaken in the School Dental Services. Information has been non-existent for some States such as NSW, which does not have a designated School Dental Service. This policy inconsistency has been reinforced by the Medicare Teen Dental Plan where eligibility for 12-17-year-olds is limited to those whose families are Family Tax A eligible. These 14-15-year-olds may be caught between receiving dental care in either the public or private system at a critical time for the development of positive oral health behaviours. For example, some New South Wales teenagers treated privately under the Medicare Teen Dental Plan have been referred back to the public sector when their parents couldn’t afford the cost of ongoing private dental care which was outside the scope of the vouchers [23].

The implementation of the Child Dental Benefits Schedule by the Australian Federal Government from 2014, provides an important opportunity to address the problem of individuals being unable to fund their private care. This scheme extends the age range of the Medicare Teen Dental Plan from 12–17 to 2–17 years and also allows a wider range of items of dental care to be provided (up to $1,000 per child over 2 years) [24]. Providers will be paid on the items provided up to the limit of $1,000, rather than a standard Medicare fee no matter what care is provided. This will also allow for improved program evaluation of the relative mix of preventive and restorative items provided.

## Conclusion

The weighted data from this survey provides the first representative information on caries experience amongst 14- and 15-year-olds in NSW. The mean DMFT score of 1.2 is lower than recent national and international data. However there are teenagers without access to fluoridated water (DMFT 1.7), living in remote areas (DMFT 2.4), and those from lower SES groups (DMFT 1.5) who have higher a prevalence of dental disease. This NSW Teen Dental Survey data should inform oral health service planners and policy makers to make informed decisions.

## Competing interests

The authors declare that they have no competing interests.

## Authors’ contributions

JS participated in the design of the study, performed the data analysis and drafted the manuscript. GJ participated in the design of the study, provided project support to the study and helped to draft the manuscript. CP participated in the design of the study, coordinated the overall study and helped to draft the manuscript. AB participated in the design of the study and helped to draft the manuscript. All authors read and approved the final manuscript.

## Pre-publication history

The pre-publication history for this paper can be accessed here:

http://www.biomedcentral.com/1471-2458/13/1060/prepub
